# Draft genome sequences of extended-spectrum β-lactamase-producing uropathogenic *Escherichia coli* strains isolated from patients with urinary tract infections

**DOI:** 10.1128/mra.00814-25

**Published:** 2025-10-07

**Authors:** Songphon Buddhasiri, Arishabhas Tantibhadrasapa, Panupon Mongkolkarvin, Chutikarn Sukjoi, Parameth Thiennimitr

**Affiliations:** 1 Faculty of Veterinary Medicine, Chiang Mai University 26682, Chiang Mai, Thailand; 2 Department of Microbiology, Faculty of Medicine, Chiang Mai University 26682, Chiang Mai, Thailand; Loyola University Chicago, Chicago, Illinois, USA

**Keywords:** uropathogenic *E coli*, urinary tract infection, ESBL

## Abstract

We report the draft genome sequences of two extended-spectrum β-lactamase-producing uropathogenic *Escherichia coli* strains, AT82 and AT84, isolated from patients with urinary tract infections in Thailand. The draft genome sizes are approximately 5,168 kb and 5,164 kb, respectively.

## ANNOUNCEMENT

Urinary tract infections (UTIs) are among the most common bacterial infections globally, with uropathogenic *Escherichia coli* (UPEC) being the predominant causative agent (1). The increasing prevalence of multidrug-resistant UPEC strains poses a growing threat to public health and clinical management (2).

In this study, two *E. coli* strains, AT82 and AT84, were isolated from urine samples of patients diagnosed with UTIs at Maharaj Nakorn Chiang Mai Hospital (MNCMH), Chiang Mai, Thailand. AT82 and AT84 were isolated from the urine collected by a Foley’s catheterization and midstream urine of 83-year-old and 54-year-old male patients with UTI admitted to MNCMH in 2020, respectively. The isolated strains were cultivated on MacConkey agar (Difco, Sparks, MD, USA) and incubated at 37°C for 18 h. The extended-spectrum β-lactamase (ESBL) production of the strains was assessed following the Clinical and Laboratory Standards Institute guidelines (3). For polymerase chain reaction (PCR) and genomic DNA isolation experiments, bacterial isolates were cultured in Luria-Bertani broth (Difco, Sparks, MD, USA) and incubated at 37°C for 16–18 h with shaking. UPEC identity was confirmed by PCR detection of four UPEC-specific marker genes (*c3509*, *c3686*, *chuA*, and *uidA*) as described by Tantibhadrasapa et al. (4). Both isolates tested positive for all four genes and were classified as UPEC strains. The study protocol was approved by the Research Ethics Committee, Faculty of Medicine, Chiang Mai University, Chiang Mai, Thailand (Approval No. MIC-2568-0350).

Bacterial genomic DNA was extracted using the DNeasy UltraClean Microbial Kit (Qiagen, Hilden, Germany) and quantified with a Qubit 2.0 Fluorometer (Thermo Fisher Scientific, USA). Whole-genome sequencing libraries were prepared using the TruSeq DNA Library Prep Kit (Illumina, San Diego, CA, USA) and sequenced on the Illumina NovaSeq platform with paired-end reads (2 × 150 bp). Raw sequencing reads were assessed with FastQC v0.11.9 (5) and processed using Trimmomatic v0.39 (6) to remove low-quality sequences and adapters (ILLUMINACLIP:TruSeq3-PE-2.fa:2:30:10:2:keepBothReads, HEADCROP:10, SLIDINGWINDOW:4:25). The genome sequences were assembled using Unicycler v0.5.1 (7), which utilized SPAdes v4.0.0 in isolate mode. Assembly quality was evaluated using QUAST v5.2.0 (8). Genome annotation was performed with the National Center for Biotechnology Information (NCBI) Prokaryotic Genome Annotation Pipeline v6.7 (9). Genome sequence information and assembly metrics for both *E. coli* strains are summarized in Table 1.

**TABLE 1:** Genome features of ESBL-producing UPEC isolates AT82 and AT84

Feature	*E. coli* strains	
	AT82	AT84
Total reads	3,654,232	4,060,051
Coverage (X, assembly-mapped)	185	172
GC content (%)	50.55	50.55
Genome size (bp)	5,168,535	5,164,838
N50 size (bp)	189,647	206,995
L50 size (bp)	10	8
Number of contigs	148	140
Completeness (%)	98.71	98.71
Predicted genes (total)	5,141	5,141
BioProject accession	PRJNA1287066	PRJNA1287066
BioSample accession	SAMN49806876	SAMN49806877
SRA accession	SRR34379154	SRR34379153
GenBank accession	JBPSNA000000000	JBPSNB000000000

## Data Availability

All data are encompassed under BioProject number PRJNA1287066. The sequencing raw reads of AT82 and AT84 were deposited in the NCBI Sequence Read Archive (SRA) accession numbers SRX29543787 and SRX29543788, respectively. The complete genome sequences of AT82 and AT84 are available https://www.ncbi.nlm.nih.gov/nuccore/JBPSNA000000000 and https://www.ncbi.nlm.nih.gov/nuccore/JBPSNB000000000, respectively.
